# Is a change in mode of travel to school associated with a change in overall physical activity levels in children? Longitudinal results from the SPEEDY study

**DOI:** 10.1186/1479-5868-9-134

**Published:** 2012-11-21

**Authors:** Lee Smith, Shannon Sahlqvist, David Ogilvie, Andy Jones, Kirsten Corder, Simon J Griffin, Esther van Sluijs

**Affiliations:** 1 MRC Epidemiology Unit, Institute of Metabolic Science, Addenbrooke’s Hospital, Box 285, Cambridge CB2 0QQ, UK; 2 UKCRC Centre for Diet and Activity Research (CEDAR), Institute of Public Health, Forvie Site, Robinson Way, Cambridge CB2 0SR, UK; 3 School of Environmental Sciences, University of East Anglia, Norwich, NR4 7JT, UK

**Keywords:** Transport, Active travel, Physical activity, School children

## Abstract

**Background:**

Children who use active modes of travel (walking or cycling) to school are more physically active than those who use passive (motorised) modes. However, less is known on whether a change in mode of travel to school is associated with a change in children’s physical activity levels. The purpose of this analysis was to investigate the association between change in mode of travel to school and change in overall physical activity levels in children.

**Methods:**

Data from 812 9–10 year old British children (59% girls) who participated in the SPEEDY study were analysed. During the summer terms of 2007 and 2008 participants completed a questionnaire and wore an accelerometer for at least three days. Two-level multiple linear regression models were used to explore the association between change in usual mode of travel to school and change in objectively measured time spent in MVPA.

**Results:**

Compared to children whose reported mode of travel did not change, a change from a passive to an active mode of travel was associated with an increase in daily minutes spent in MVPA (boys: beta 11.59, 95% CI 0.94 to 22.24; girls: beta 11.92, 95% CI 5.00 to 18.84). This increase represented 12% of boys’ and 13% of girls’ total daily time spent in MVPA at follow-up.

**Conclusion:**

This analysis provides further evidence that promoting active travel to school may have a role in contributing to increasing physical activity levels in children.

## Background

Physical activity is known to be positively associated with overall health in children [1,2]. Active travel provides an opportunity for children to incorporate physical activity into their daily lives and has been shown to be associated with health outcomes. For example, a systematic review identified nine high quality studies of the association between walking and cycling (active travel) to school and weight status or body composition in young people, of which 55% demonstrated a significant inverse association [3]. However, the proportion of UK children walking to school has decreased since 1995 and now stands at less than 50% [4].

Cross-sectional studies identified in systematic reviews suggest that young people who walk or cycle to school can accumulate between 5 and 37 additional minutes per day (min/day) of moderate-to-vigorous physical activity (MVPA) compared to those who use motorised (passive) modes of transport [5,6]. Associations between walking or cycling to school and physical activity have been shown to differ by sex, although the evidence is inconsistent. For example, Rosenberg et al. found stronger associations in boys than in girls [7] whereas Cooper et al. found an association only in girls [8]. Using cross-sectional data from the *Sport Physical Activity and Eating Behaviour: Environmental Determinants in Young People (SPEEDY)* study, Panter et al. found that girls and boys who walked to school spent an additional 6.9 min/day and 3.3 min/day, respectively, in overall MVPA compared to those who used passive modes of transport. Girls and boys who cycled to school spent an additional 3.7 min/day and 6.5 min/day, respectively, in overall MVPA [9].

These findings suggest that active travel is associated with higher overall levels of physical activity [5,6]. However, given their cross-sectional nature it is difficult to establish the direction of causality. More importantly from a public health perspective, it remains unclear whether changing from a passive to an active mode of travel is necessarily associated with an increase in overall physical activity. It is possible that children might compensate for a change to an active mode of travel with a decrease in other forms of physical activity (activity compensation) [10,11]. On the other hand, a previous study investigated the association between taking up cycling to school and cardiorespiratory fitness over a six-year follow-up showed that taking up cycling to school was a significant predictor of increased cardiorespiratory fitness at follow-up [12]. One possible explanation is that taking up cycling to school may have increased participants’ overall physical activity levels and thus cardiorespiratory fitness. Alternatively participants who became more physically active in general between baseline and follow-up, and therefore improved their cardiorespiratory fitness, may then have taken up cycling to school.

In order to assess the potential role of active travel for increasing population levels of physical activity, it is important to investigate whether the above association can be found when using physical activity rather than fitness as an outcome. We therefore investigated the association between change in mode of travel to school and change in objectively measured physical activity over a one-year period in 9–10 year old British school children taking part in the SPEEDY study.

## Methods

Full details of participant recruitment and study procedures are provided elsewhere [13,14]. In brief, the SPEEDY study is a prospective population-based cohort study examining factors associated with physical activity and dietary behaviour in pupils aged 9–10 years (at baseline) sampled through schools in the county of Norfolk, UK. A total of 157 schools were approached, of which 92 (59%) agreed to participate. All Year 5 (9–10 years old) pupils in these schools (n=3619) were invited and a participation rate of 57% (n=2064) was achieved. Two waves of data collection were carried out during the summer terms (April to July) of 2007 and 2008. At baseline, both pupils and their parents were asked to complete separate questionnaires about the pupils’ dietary and physical activity behaviour. Pupils were also asked to complete an additional questionnaire at follow-up. At both time points pupils were asked to wear an Actigraph accelerometer (GTIM, Actigraph LCC, Pensacola, US) for seven consecutive days. Accelerometers were programmed to store activity data at five second intervals. Questionnaires and accelerometers were administered at the same point in time at baseline (by research staff directly) and at follow-up (by post) in an attempt to maximise coherence between measures. Written parental consent was obtained prior to data collection and ethical approval was granted by the University of East Anglia research ethics committee.

### Exposure measure

To assess travel behaviour, pupils answered the same question at both time points: ‘How do you usually travel to school?’ Children were asked to indicate the main travel mode used: (a) car, (b) bus or train, (c) walk, or (d) bike (cycle). Use of the car, bus, or train was defined as ‘passive travel’ and walking and cycling were defined as ‘active travel’, consistent with other studies of the relationship between travel mode and physical activity [7,8]. Depending on their responses to this question at both time points, pupils were categorised into one of four exposure categories: 1) ‘consistent active travel’, 2) ‘consistent passive travel’, 3) ‘change from passive to active travel’ and 4) ‘change from active to passive travel’.

### Outcome measures

Accelerometer data were cleaned using a standard program (available at http://www.mrc-epid.cam.ac.uk). All data collected between 2300 and 0600 hours were removed as well as data from the first day (a partial wear day) because of the wide range of times at which participants received their accelerometer. Ten minutes of continuous zero counts were classified as ‘non-wear time’, which amounted to an average of five hours of non-wear time per participant per day. Accelerometer data were summarised as time (min/day) spent in MVPA where MVPA was defined as ≥2000 counts per minute, a cut point applied in previous studies in this age group [15]. To explore any differences in physical activity levels by weekdays and weekends, time spent in MVPA was aggregated into the following two summary periods: (i) weekday MVPA (Monday to Friday between 0600 and 2300) and (ii) total daily MVPA (weekdays and weekend days between 0600 and 2300). To be included in the weekday analysis, three or more valid weekdays (wear time ≥500 min/day) of data were required at both baseline and follow-up. To be included in the total daily analysis, three or more valid weekdays or weekend days of data were required at both baseline and follow-up. The choice of a three-day criterion reflected adequate reliability of the accelerometry measures across three or more valid wear days in this dataset (intraclass correlation coefficient = 0.78).

To assess whether change in mode of travel between baseline and follow-up was associated with change in physical activity, and to allow results to be expressed in a meaningful metric, the difference in the number of minutes spent in MVPA between baseline and follow-up was calculated for each participant for both weekday and total daily analysis. To account for any difference in accelerometer wear time between the two measurement periods, the change in time spent in MVPA was calculated for every 1000 minutes of wear time as follows: (1-year follow-up MVPA x 1000/1-year follow-up wear time) – (baseline MVPA x 1000/baseline wear time). The constant of 1000 was chosen as it is approximately equal to the highest observed daily accelerometer wear time in the dataset (1053 min) and corresponds to a reasonable maximum realistic daily wear time in this age group (~17 hours).

### Covariates

At baseline pupils reported their sex and age, and their parent or main carer reported household car ownership and their own highest educational qualification (in categories). Objective measures of urban/rural status and network distance from home to school (based on home postcode) were estimated using a Geographic Information System [16]. Trained research assistants measured pupils’ height and weight (whilst children wore light clothing) according to standard operating procedures from which body mass index (BMI) was calculated in kg/m^2^. These covariates were selected because they were hypothesised to be independently associated with both the exposure (change in travel behaviour) and the outcome (change in physical activity).

### Analysis

Characteristics of the study population at baseline and follow-up were summarised using descriptive statistics. To assess whether change in mode of travel was associated with change in physical activity, two-level linear regression models were used with pupils at level 1 and school at level 2, to account for the clustering of outcomes amongst children within the same schools. The outcomes were change in average weekday time spent in MVPA and change in average total daily time spent in MVPA (computed as described above). The exposure was change in travel mode (categorised as described above). Next, pre-specified covariates as well as baseline time spent in MVPA (min/day) were entered into the models as potential confounders. To account for the well documented sex differences in the association between physical activity and active travel, [5,6] analyses were also stratified by sex.

## Results

A total of 2064 pupils took part at baseline, of whom 55.1% were girls and 96.2% were white. Of the 1019 pupils who took part at follow-up, 954 returned an activity monitor. 812 pupils were entered into the weekday analyses owing to incomplete or missing MVPA (n=96) or travel mode (n=46) data. The same 812 pupils were entered into total daily (weekdays plus weekend days) analyses. After excluding cases with missing data for potential confounders (predominantly parental education), final adjusted models included 772 pupils. There were no significant differences between the characteristics (sex, ethnicity, BMI, urban/rural status, parental education or baseline MVPA: all p>0.05) of pupils who participated at baseline only (n=1244) and those included in the final adjusted models (n=772). 59% of pupils included in analyses were girls and 97% were white.

Table 1 shows participant demographics and frequency of travel mode at baseline and follow-up. At both time points, most pupils either walked or were driven to school. 40.6% (n=330) of pupils were categorised as reporting ‘consistent passive travel’ and 44.1% (n=358) as reporting ‘consistent active travel’. 6.1% of pupils (n=50) were categorised as having changed from an active to a passive mode of travel, and 9.2% (n=75) from a passive to an active mode.

**Table 1:** Participant demographics and travel behaviour at baseline and follow-up

**Variable**	**Frequency in complete baseline sample**		**Frequency in complete 1-year follow-up sample**		**Frequency at 1-year follow-up among those who had valid MVPA and travel mode data**	
	**N (%)**		**N (%)**		**N (%)**	
**Sex**	**n**=2064		**n**=913		**n**=812	
Boy	926	(45)	374	(41)	333	(41)
Girl	1,138	(55)	539	(59)	479	(59)
**Ethnicity**	**n**=1914		**n**=885		**n**=794	
White	1841	(96)	857	(97)	770	(97)
Other	73	(4)	28	(3)	24	(3)
**Urban/rural status**	**n**=2023		**n**=902		**n**=802	
Hamlet and isolated dwelling	142	(7)	73	(8)	67	(8)
Village	506	(25)	248	(28)	219	(27)
Town and fringe	575	(28)	275	(30)	244	(30)
Urban >10 k	800	(40)	306	(34)	272	(34)
**Parent education status**	**n**=1882		**n**=877		**n**=786	
None	140	(7)	53	(6)	45	(6)
GCSE or equivalent	963	(51)	454	(52)	405	(51)
A-levels or equivalent	468	(25)	204	(23)	186	(24)
Degree or postgraduate degree	311	(17)	166	(19)	150	(19)
**Travel mode**	**n=**2053		**n**=913		**n**=812	
Bicycle	189	(9)	73	(8)	59	(7)
Bus or train	127	(6)	62	(7)	57	(7)
Car	923	(45)	357	(39)	322	(40)
Foot	814	(40)	421	(46)	374	(46)

Pupils who changed their usual mode of travel did not differ significantly from the total study sample in terms of sex, ethnicity or home location. Table 2 shows that pupils who changed to an active mode of travel and those who changed to a passive mode recorded a mean total daily increase and decrease respectively in minutes spent in MVPA. Table 3 shows similar findings for change in mean weekday minutes spent in MVPA.

**Table 2:** Unadjusted mean (SD) changes in total daily minutes spent in MVPA from baseline to follow-up

**Change in mode of travel to school between baseline and one-year follow-up**	**Boys (n=332)**		**Girls (n=480)**	
	**Total daily time spent in MVPA at baseline**	**Change in total daily time spent in MVPA**^**a**^	**Total daily time spent in MVPA at baseline**	**Change in total daily time spent in MVPA**^**a**^
**Active to passive (n=50)**	84 (22)	−17 (81)	67 (14)	−6 (22)
**Passive to active (n=75)**	78 (29)	9 (31)	45 (17)	6 (29)
**Consistent active (n=358)**	84 (23)	−5 (27)	66 (26)	−6 (25)
**Consistent passive (n=330)**	84 (24)	−5 (31)	67 (20)	−4 (26)

n=812, ^a^ time spent in MVPA is expressed as (1-year follow-up MVPA x 1000/1-year follow-up wear time) – (baseline MVPA x 1000/baseline wear time).

**Table 3:** Unadjusted mean (SD) changes in weekday minutes spent in MVPA from baseline to follow-up

**Change in mode of travel to school between baseline and one-year follow-up**	**Boys (n=332)**		**Girls (n=480)**	
	**Weekday time spent in MVPA at baseline**	**Change in weekday time spent in MVPA**^**a**^	**Weekday time spent in MVPA at baseline**	**Change in total daily time spent in MVPA**^**a**^
**Active to passive (n=50)**	82 (29)	−12 (20)	68 (19)	−4 (24)
**Passive to active (n=75)**	80 (24)	8 (28)	65 (16)	10 (28)
**Consistent active (n=358)**	82 (23)	1 (27)	66 (20)	−3 (24)
**Consistent passive (n=330)**	83 (23)	−1 (30)	66 (18)	0 (22)

n=812, ^a^ time spent in MVPA is expressed as (1-year follow-up MVPA x 1000/1-year follow-up wear time) – (baseline MVPA x 1000/baseline wear time).

In two-level multiple linear regression analyses (Tables 4 and 5), change to an active mode of travel was positively associated with an increase in weekday and total daily time spent in MVPA compared with the reference category of ‘consistent active travel’. This association remained after adjustment for potential confounders and was observed in boys and girls separately, with the exception of weekday MVPA for boys for which no significant association was found. No significant associations were found for other change in travel mode groups.

**Table 4:** Associations between change in mode of travel to school and change in mean total daily minutes spent in MVPA

**Variable**	**Children (n=772)**					**Boys (n=314)**					**Girls (n=458)**				
	**Coefficient**	**95% CI**			**P**	**Coefficient**	**95% CI**			**P**	**Coefficient**	**95% CI**			**P**
**Change in mode of travel (reference: consistent active)**															
Active to passive	−3.12	−10.26	to	4.02	0.39	−12.31	−24.98	to	0.36	0.05	1.39	−6.98	to	9.76	0.74
Passive to active	12.08	6.14	to	18.02	<0.01	11.78	1.16	to	22.41	0.03	11.92	5.01	to	18.84	<0.01
Consistent passive	1.98	−1.63	to	5.60	0.28	1.27	−4.95	to	7.50	0.68	2.76	−1.56	to	7.10	0.21
**Sex (reference: girl)**															
Boy	10.75	7.11	to	14.40	<0.01										
**Parental education (reference: none)**															
GCSE or equivalent	7.56	0.21	to	14.90	0.04	3.73	−9.85	to	17.33	0.59	11.01	2.54	to	19.47	0.01
A level or equivalent	3.95	−3.81	to	11.72	0.31	−1.49	−15.89	to	12.90	0.83	8.19	-.69	to	17.09	0.07
University/postgraduate degree	4.99	−3.09	to	13.08	0.22	3.83	−10.77	to	18.44	0.60	6.32	−3.19	to	15.84	0.19
**Location (reference: hamlet and isolated dwelling)**															
Village	0.09	−6.71	to	6.91	0.97	−4.74	−15.73	to	6.24	0.39	3.33	−5.26	to	11.93	0.44
Town and fringe	−6.07	−7.70	to	6.48	0.86	0.06	−10.97	to	11.10	0.99	-.68	−9.87	to	8.50	0.88
Urban >10 k	−2.61	−9.28	to	4.06	0.44	−6.01	−16.46	to	4.43	0.26	0.77	−7.90	to	9.45	0.86
**Age (years)**	2.93	−2.68	to	8.56	0.30	1.05	−8.98	to	11.09	0.83	3.88	−2.74	to	10.52	0.25
**BMI**	-.30	-.87	to	0.25	0.28	-.53	−1.67	to	0.61	0.36	−1.05	-.72	to	0.51	0.73
**Route length (metres)**	-.00	-.00	to	0.00	0.15	-.00	-.00	to	0.00	0.48	-.00	-.00	to	0.00	0.25
**Car ownership (reference: no car)**															
Owns one car	−4.99	−14.20	to	4.21	0.28	6.24	−8.85	to	21.35	0.41	−16.79	−28.52	to	−5.06	<0.01
Owns more than one car	−6.76	−15.91	to	2.38	0.14	5.10	−9.74	to	19.96	0.50	−19.09	−30.81	to	−7.38	<0.01
**Baseline time spent in MVPA (min/day)**	-.67	-.74	to	-.59	<0.01	-.67	-.79	to	-.55	<0.01	-.66	-.76	to	-.56	<0.01

95% CI: 95% confidence interval.

**Table 5:** Associations between change in mode of travel to school and change in mean weekday minutes spent in MVPA

**Variable**	**Children (n=772)**					**Boys (n=314)**					**Girls (n=458)**				
	**Coefficient**	**95% CI**			**P**	**Coefficient**	**95% CI**			**P**	**Coefficient**	**95% CI**			**P**
**Change in mode of travel (reference: consistent active)**															
Active to passive	−4.63	−12.19	to	2.92	0.23	−15.12	−28.52	to	−1.72	0.02	1.08	−7.74	to	9.91	0.80
Passive to active	10.81	4.51	to	17.11	<0.01	7.66	−3.65	to	18.97	0.18	11.95	4.67	to	19.24	<0.01
Consistent passive	1.96	−1.87	to	5.79	0.31	0.28	−6.28	to	6.84	0.93	3.78	-.78	to	8.36	0.10
**Sex (reference: girl)**															
Boy	4.99	1.12	to	8.86	0.01										
**Parental education (reference: none)**															
GCSE or equivalent	8.60	0.80	to	16.40	0.03	0.46	−14.20	to	15.13	0.95	13.85	4.91	to	22.79	<0.01
A level or equivalent	5.02	−3.20	to	13.26	0.23	−5.62	−21.11	to	9.86	0.47	11.91	2.53	to	21.29	0.01
University/postgraduate degree	7.40	−1.18	to	16.00	0.09	2.19	−13.58	to	17.98	0.78	10.51	0.47	to	20.56	0.04
**Location (reference: hamlet and isolated dwelling)**															
Village	−1.15	−8.48	to	6.16	0.75	−1.80	−13.85	to	10.24	0.76	0.94	−8.14	to	10.04	0.83
Town and fringe	−1.27	−9.12	to	6.58	0.75	2.79	−9.59	to	15.18	0.65	−3.92	−13.75	to	5.89	0.43
Urban >10 k	-.23	−7.37	to	6.90	0.94	-.47	−11.72	to	10.78	0.93	1.62	−7.55	to	10.81	0.72
**Age (years)**	1.24	−4.75	to	7.23	0.68	0.86	−9.80	to	11.53	0.87	0.64	−6.36	to	7.66	0.85
**BMI**	-.36	-.96	to	.22	0.22	-.17	−1.39	to	1.05	0.78	-.37	−1.02	to	0.27	0.26
**Route length (metres)**	-.00	-.00	to	.00	0.65	0.00	-.00	to	0.00	0.87	-.00	-.00	to	0.00	0.42
**Car ownership (reference: no car)**															
Owns one car	1.60	−8.14	to	11.36	0.74	6.39	−9.73	to	22.53	0.43	−5.36	−17.75	to	7.02	0.39
Owns more than one car	0.12	−9.58	to	9.82	0.98	4.55	−11.37	to	20.49	0.57	−6.98	−19.36	to	5.39	0.26
**Baseline time spent in MVPA (min/day)**	-.27	-.35	to	-.18	<0.01	-.27	-.39	to	-.14	<0.01	-.26	-.37	to	-.15	<0.01

95% CI: 95% confidence interval.

A total of 34 participants included in the analysis moved home between baseline and follow-up. These participants were all in the ‘consistent active travel’ (n=18) or ‘consistent passive travel’ (n=16) groups. Excluding these participants from the analyses made no substantive difference to the findings (results not shown).

## Discussion

In this population-based longitudinal study of 9–10 year old children, boys and girls who changed to an active mode of travel increased their total daily MVPA by an average of 9 min and 6 min respectively. This represents 12% of boys’ and 13% of girls’ total daily time spent in MVPA at follow-up. The total daily MVPA of children who did not change their travel mode, or who changed to a passive mode, decreased over the same period. It should be noted however that boys and girls who changed to an active mode spent less time in MVPA (6 min/day and 22 min/day, respectively) at baseline than children in the other exposure categories and may therefore have had greater potential to increase their physical activity levels. However, models were adjusted for students’ baseline time spent in MVPA, to control for potential regression to the mean. The results presented here, although using a more proximal outcome, support previous work by Cooper et al. who found that boys and girls who took up cycling to school during a 6-year period had better cardiorespiratory fitness than those who did not cycle to school at either baseline or follow-up [12].

For boys who changed to an active mode of travel, no significant association was observed with time spent in MVPA on weekdays. However, a significant association was observed with daily time spent in MVPA over the entire week. These findings suggest that the observed association between active travel and overall physical activity may reflect the *combined* effect of the journeys themselves, spontaneous play during the journeys and an encouragement of active behaviour in other areas of children’s lives [6]. It is also possible that increases in active travel may reflect a change in travel mode preference resulting from a more general increase in physical activity; in other words, children who become more active in general may then choose to travel to school by active means. Further analyses using multiple time points are needed to investigate these potential causal pathways.

Children who consistently used either an active or a passive mode of travel recorded decreases in MVPA over the one-year study period, as did children who changed from an active to a passive mode. This suggests that while taking up an active mode of travel may negate the downward trend in MVPA that is typically seen in this age group [14,17], continuing active travel is not sufficient to do so. The decline in MVPA seen among children who remained active travellers highlights a need for interventions that prevent a decline in physical activity in other areas of children’s lives, for example during and after school, as well as interventions to promote active travel.

In this study only 9.2% of pupils changed to an active mode of travel, while 40.6% consistently used passive modes. In 2005, 43% of UK children were driven to school [4]. Interventions that promote active modes of travel to school therefore have the potential to increase the activity levels of large numbers of pupils, which may have important public health implications [1,18]. However, a recent systematic review found that to date, interventions promoting active travel to school have produced only small effects, suggesting a need for further research as to how best to achieve such behaviour change in practice [19].

Two-level regression models showed that parental education — an indicator of socioeconomic status (SES) — predicted changes in girls’ time spent in MVPA but not boys’. Since boys participate in more active play in their free time than girls [20], it is possible that parents encourage their daughters, or girls choose, to accumulate MVPA in other ways such as organised sports. The costs associated with participating in organised sports (such as those of equipment and membership fees) might contribute to explaining why parental SES appears to be an important influence on girls’ time spent in MVPA and not associated with boys’.

### Strengths and limitations

Strengths of this study include its prospective design, the use of objective measures of physical activity and that it was conducted in a large population-based sample of UK primary school children. The one year interval separating baseline and follow-up data collection ensured seasonally matched data, thereby reducing the risk of artefactual changes in either usual travel behaviour or measured physical activity attributable to different weather conditions at the two time points. It also provided sufficient time to result in changes to children’s usual travel behaviour. However, despite the prospective design, it remains unclear whether children become more physically active and then changed to an active mode of travel, or vice versa. A longitudinal dataset including measurement at three or more time points or a controlled intervention study would be needed to answer this question with confidence.

Although the analysis sample comprised approximately one-third of the pupils recruited at baseline, we found no significant differences between the characteristics (sex, BMI, urban rural/status, parental education or baseline MVPA) of pupils included in and excluded from the analysis. However, we have previously shown that in comparison to the Norfolk population a smaller proportion of obese children took part in the SPEEDY study, and that the demographic profile of the county of Norfolk (with only 3.8% non-white children) is not representative of the whole of the UK [13].

The measure of travel mode was relatively crude and did not allow multi-modal trips or the daily breakdown of travel behaviour to be ascertained. Moreover, to our knowledge there is no published validation of questions to ascertain ‘usual travel mode’ in children. However, similar measures have been used in previous studies examining the association between travel mode and physical activity in children [8,21]. Furthermore, an insufficient proportion (6%) of pupils used public transport at baseline to allow public transport to be treated as a separate category. Consequently, if we had disaggregated travel mode further into car, public transport, walking and cycling our statistical models would had been underpowered to detect associations. As in previous studies, public transport was therefore combined with passive travel [22]. Although travelling by public transport usually includes some walking, school bus services in the UK often pick up pupils close to their homes and it is therefore reasonable to assume that these journeys might have included relatively little active travel. In support of this assumption, van Sluijs et al. found that UK children who walked to school had significantly higher average weekly accelerometer counts per minute and total minutes of MVPA than those classified as using the car or public transport to travel to school [23]. Moreover, removing pupils who used public transport from the current analyses made no substantive differences to the findings (results not shown). Distance from home to school has consistently been found to be associated with mode of travel to and from school [24,25]. Although network distance was included as a covariate in analysis, it was not possible to stratify the analysis by this measure owing to small cell sizes.

Accelerometers as used in this study are calibrated to record ambulatory activity (hip movement) and therefore underestimate the intensity of physical activity undertaken whilst cycling. In this study only 9% and 7% of pupils reported cycling to school at baseline and follow-up respectively, so the influence of this limitation of measurement is likely to be small. Criteria for classifying accelerometer non-wear time vary within the literature [26,27]. For the present analysis 10 minutes of continuous zero counts were used to define non-wear. It appears reasonable to assume that 9–10 year old children are unlikely to remain sufficiently still to register no movement on an accelerometer for more than 10 minutes when awake, and this criterion has been applied in previous studies [26,28].

## Conclusion

Taking up active travel to school is associated with increased levels of MVPA in primary school children. This study provides further evidence that promoting active travel to school may have a role in contributing to increasing physical activity levels in children.

## Competing interest

The authors declare that there are no conflicts of interest.

## Authors’ contributions

A.J, K.C, S.J.G and E.S designed the SPEEDY study and coordinated its implementation. L.S conducted the statistical analyses and drafted the manuscript. A.J, K.C, S.J.G, E.S, D.O and S.S assisted in interpreting the statistical analyses and drafting the manuscript. All authors read and approved the final manuscript.
